# Evolution of a Novel Business Leadership Course for General Surgery Chief Residents

**DOI:** 10.7759/cureus.3559

**Published:** 2018-11-08

**Authors:** Adham R Saad, Clinton E Baisden, Daniel L Dent, Leen Khoury, Melissa Panzo, Stephen M Cohn

**Affiliations:** 1 Surgery, University of South Florida, Tampa, USA; 2 Surgery, University of Texas Health Science Center, San Antonio, USA; 3 Surgery, Staten Island University Hospital, Staten Island, USA; 4 Emergency Medicine, Staten Island University Hospital, Staten Island, USA

**Keywords:** curriculum, business education, practice management, leadership

## Abstract

Background

General surgery chief residents are typically well equipped for board examinations but poorly trained to deal with the business challenges of surgical practice. We began a business leadership course to better prepare them for their careers.

Methods

Chief residents were given one-hour lectures with topics that included: Differences between private/academic practice, personal finances, contracts, practice management, legal issues and health law, and time management.

Results

Initial evaluations revealed that the topics covered and the presentations were well received. Subsequently, the course was moved to earlier in the academic year to prepare them for contract negotiations and then to Sunday nights to decrease interruptions and allow spouse participation.

Conclusions

The course evolved into a program that the chief residents feel is an important addition to their education. Moving the meetings to a weekend evening improved attendance, decreased interruptions, and allowed participation by spouses and significant others.

## Introduction

Traditionally, surgical training programs have focused on trainees attaining relevant technical skills and clinical acumen [1]. Surgical mentors have invested little of their time on educating residents on how to navigate the healthcare landscape. Physicians must now be equipped to take an active leadership role and have a solid business knowledge base to interact optimally with our complex healthcare system.

Lusco and colleagues [2] found that the great majority of general surgery program directors (87%) agreed that residents should be formally trained in business management. Only 34% of surgery programs currently offer exposure to business management and 42% include just three hours or less per year of training. Additionally, program directors felt that the 80-hour work week restrictions made it even more difficult to add a business management arm to their education.

Because of a perceived need to educate our residents in the business of medicine, we established a new lecture series for our chief residents as part of a comprehensive business leadership course. Its inception and evolution are the subject of this report.

## Materials and methods

Initially, lecture topics were chosen by two of the authors (Stephen Cohn and Daniel Dent) according to the perceived needs of general surgery chief residents at the University of Texas Health Science Center at San Antonio. Speakers from the medical school or local community who were identified as experts in a specified field were asked to donate time and effort to give a specific presentation. The presentations were scheduled early in the morning on the department’s academic day, weekly, following the American Board of Surgery In-Training Examination (ABSITE) exam. The following year, the seminars were moved to monthly throughout the academic year.

Records of each lecture were kept, indicating its date, time, location, title, speaker’s name, and attendance. Following each lecture, confidential evaluations using a five-point scale from worst (1) to best (5) were anonymously obtained from each attendee, rating the overall impression, choice of topic, speaker effectiveness, and use of audiovisual aids. It was anticipated that these evaluations would be used for process improvement and direct changes in the lecture series with time. To this end, the attendees were also provided space on the evaluation forms to give opinions regarding the following three additional questions: (1) What one thing, if any, did the attendee especially like about the lecture?, (2) What one thing, if any, could have made it better?, and (3) Any additional comments you wish to add?

Finally, chief resident attendance records were obtained from the surgery department to determine how many chief residents could not have been present due to vacations, meetings, or other official business. If on vacation or other official absence, they were not included in calculating attendance.

## Results

There were nine lectures given during the first series, which occurred between February and May of 2005. Seven lectures occurred during the second year and five had occurred by the time this report was prepared during the third year.

Year 1

In the first year, business leadership course (BLC) lectures were given weekly. Recorded attendance appeared good but actual attendance suffered from multiple interruptions due to pagers, telephone calls, and emergencies. Attendee evaluations revealed that the topics covered and presentations were considered helpful. Residents commented that the course would be better if offered monthly and needed to be moved earlier in the training year to prepare chief residents for contract negotiations. It was suggested that spouses or significant others should be invited since many were interested. Evaluations of two presenters were low, and they were not invited back.

Year 2

In the second year, presentations were changed to a monthly basis and moved to earlier in the year. Spouses and significant others were invited; however, none attended, stating Monday mornings interfered with work and/or childcare responsibilities. Some speakers and topics were changed with a slight improvement in evaluations although interruptions remained frequent. Comments were that more time needed to be devoted to personal finance, legal issues, and time management. It was decided that the lecture series needed to be moved away from the medical center and scheduled at a more available day and time.

Year 3

In the third year, lectures were moved to Sunday evening dinner parties to allow the participation of significant others and to reduce interruptions. The residents and their spouses or guests met at a senior faculty home at 6:30 PM for social interaction followed by dinner at 7:00 PM. The lecture and discussion lasted from 7:30 PM to 8:30 PM, followed by dessert and departure by 8:45 PM. Babysitting was provided in the host home by one or two faculty family volunteers.

This format proved to be highly successful in improving attendance, especially among spouses and significant others and seemed to greatly reduce interruptions. Whereas chief resident attendance was maintained at 87%, 78%, and 90% in years one to three, spouse or significant other attendance increased from zero in Year 2 to 83% in Year 3 (p<.01). Overall lecture evaluations improved from a mean of 4.19 to 4.32 to 4.48 (1 to 5 scale) in Years 1 to 3 (Table 1).

**Table 1:** Lecture evaluations Evaluation (Eval); Year (YR)

Attendance	Attendance	# Possible	General	Speaker	Topic	Aids	Overall	Lecture Name
Chiefs %	Spouse %	Attendees	Eval	Eval	Eval	Eval	Eval	
89	n/a	9	n/d	n/d	n/d	n/d	n/d	Difference between private and academic practice
n/a	n/a	n/d	n/d	5	5	n/d	5	What makes a successful private practice
89	n/a	9	n/d	3.5	3.8	n/d	3.7	Finances and contracts
75	n/a	8	n/d	2	2.3	n/d	2.15	Contracts
100	n/a	9	n/d	4	4	n/d	4	What to do when looking for a job
78	n/a	9	n/d	4	5	n/d	4.5	How to succeed in academics
100	n/a	6	n/d	5	5	n/d	5	Legal issues, informed consent, asset protect
75	n/a	8	n/d	5	5	n/d	5	Personal finance
87%	n/a		n/d	4.1	4.3	n/d	4.19	AVERAGE YR. 1
YEAR 2								
100	0	8	5	5	5	4.8	4.95	Personal finance
100	0	5	3.5	3.5	3.5	2.75	3.3	Practice management
89	0	9	4.4	4.4	4.4	4.3	4.4	How to find a job
44	0	9	5	5	5	5	5	What makes a successful private practice
n/a	0	n/d	n/d	n/d	n/d	n/d	n/d	Finances and contracts
n/a	0	n/d	n/d	n/d	n/d	n/d	n/d	Legal issues, informed consent, asset protect
56	0	9	4	4.2	4.6	3	3.95	Difference between private and academic practice
78%	0		4.38	4.42	4.5	3.97	4.32	AVERAGE YR. 2
YEAR 3								
100	100	5	5	4.56	5	4.75	4.95	Personal finance I
80	60	5	5	5	5	5	5	Personal finance II
100	100	6	3.9	3.9	4.4	2.5	3.67	Time management
86	86	7	4.7	4.55	4.55	4.4	4.55	Economics of surgical practice
86	71	7	4.2	4.3	4.7	3.8	4.25	What I learned & wished I'd learned
90%	83		4.56	4.56	4.73	4.09	4.48	AVERAGE YR. 3

## Discussion

Residents at all levels of training have described many areas in leadership and business where they felt uncomfortable, including billing, coding, and risk management [3]. As Chassin et al. [4] purport, physicians that do not have adequate training in the business aspects of practice find themselves spending the majority of their time learning the basics of managing a successful practice. It has been the unfortunate tendency of the American medical community to neglect its leadership role within the healthcare industry, with possible harm to patient care and the entire healthcare system. Schwartz [5] implores this is due to the fact that the “initial and subsequent development of physician leadership has occurred at random” and that to remedy this problem, “[physician leadership] must become an organized and conscious effort on the part of surgical educators in order that physicians may again lead the American health system.”

Surgery departments around the country have been required to develop curriculums that accommodate the 80-hour work week while continuing to maintain adequate educational time. This has been a significant obstacle to instituting leadership programs for surgical residents. Surgical residents must have strong time management skills and feel that this is one of the most important traits for their success. Fortunately, greater than 75% of surgical residents deemed themselves competent in time management [1].

Despite this perceived strength, surgical residents have difficulty balancing clinical and educational responsibilities with their commitments outside the hospital, as evidenced by the results of our study. Through the first year of the study, when the leadership lectures were held on Monday mornings as an hour addendum to the educational curriculum, attendance suffered secondary to multiple interruptions due to beepers, phone calls, and clinical emergencies. Through feedback, residents suggested that their spouses be invited to the lectures so that they would have insight into the profession, an understanding of the business model, and a reasonable expectation of the future. Therefore, spouses were invited to attend the Monday morning lectures, but the scheduling of the lectures was unreasonable for the spouses. None could attend secondary to childcare issues and professional commitments.

The most significant adaptation we made to the curriculum was moving the meetings from Monday morning to Sunday evenings. This move was accompanied by a change in setting from the university to a more informal setting at a faculty’s residence. A key benefit to this change in setting was the ability to provide childcare during the lectures and discussion. These changes provided a significant increase in spousal attendance. In addition, according to the evaluations, the residents found the lectures to be much more enjoyable and productive. The informal setting allowed for better and more comfortable discussions. The change to Sunday evenings also provided childcare opportunities, allowing for the spouses to attend the meeting without much difficulty.

## Conclusions

Our business leadership course has evolved into a program that our chief residents, their significant others, and the participating faculty feel is an important addition to their education. Moving the meetings from a weekday morning to Sunday evenings improved attendance, decreased interruptions, and allowed participation by significant others. We hope to continue to make improvements to our leadership curriculum. Future plans include expanding the program to include postgraduate year (PGY) 3 and 4 residents in addition to the chief residents to provide a more complete curriculum for our residents prior to their graduation.
